# MALAT1 shuttled by extracellular vesicles promotes M1 polarization of macrophages to induce acute pancreatitis via miR‐181a‐5p/HMGB1 axis

**DOI:** 10.1111/jcmm.16844

**Published:** 2021-08-27

**Authors:** Jie Liu, Zequn Niu, Rui Zhang, Zhuo Peng, Liming Wang, Zhong Liu, Yanxia Gao, Honghong Pei, Longfei Pan

**Affiliations:** ^1^ Department of Emergency Medicine The Second Affiliated Hospital of Xi’an Jiaotong University Xi’an China

**Keywords:** acute pancreatitis, extracellular vesicles, high‐mobility group box 1 protein, long non‐coding RNA, M1 polarization of macrophages, metastasis‐associated lung adenocarcinoma transcript‐1, microRNA‐181a‐5p

## Abstract

Acute pancreatitis (AP) is a serious condition carrying a mortality of 25–40%. Extracellular vesicles (EVs) have reported to exert potential functions in cell‐to‐cell communication in diseases such as pancreatitis. Thus, we aimed at investigating the mechanisms by which EV‐encapsulated metastasis‐associated lung adenocarcinoma transcript‐1 (MALAT1) might mediate the M1 polarization of macrophages in AP. Expression patterns of MALAT1, microRNA‐181a‐5p (miR‐181a‐5p) and high‐mobility group box 1 protein (HMGB1) in serum of AP patients were determined. EVs were isolated from serum and pancreatic cells. The binding affinity among miR‐181a‐5p, MALAT1 and HMGB1 was identified. AP cells were co‐cultured with EVs from caerulein‐treated MPC‐83 cells to determine the levels of M1/2 polarization markers and TLR4, NF‐κB and IKBa. Finally, AP mouse models were established to study the effects of EV‐encapsulated MALAT1 on the M1 polarization of macrophages in AP in vivo. MALAT1 was transferred into MPC‐83 cells via EVs, which promoted M1 polarization of macrophages in AP. MALAT1 competitively bound to miR‐181a‐5p, which targeted HMGB1. Moreover, MALAT1 activated the TLR4 signalling pathway by regulating HMGB1. EV‐encapsulated MALAT1 competitively bound to miR‐181a‐5p to upregulate the levels of IL‐6 and TNF‐α by regulating HMGB1 via activation of the TLR4 signalling pathway, thereby inducing M1 polarization of macrophages in AP. In vivo experimental results also confirmed that MALAT1 shuttled by EVs promoted M1 polarization of macrophages in AP via the miR‐181a‐5p/HMGB1/TLR4 axis. Overall, EV‐loaded MALAT1 facilitated M1 polarization of macrophages in AP via miR‐181a‐5p/HMGB1/TLR4, highlighting a potential target for treating AP.

## 1 INTRODUCTION

Acute pancreatitis (AP) is a type of pancreatic inflammatory disease with sudden onset, and its incidence varies in different regions^1^ with more females suffered from it than males.^2^ The fatality rate of AP is closely related to the frequency and pathogenesis of the disease, and the fatality rate of first‐episode AP is 14 times that of recurrent pancreatitis.^3^ Gallstones and alcohol abuse are the key factors for AP, and the fatality rate of AP with biliary stones is twice higher than that of AP with alcoholism.^4^ In addition, genetic factors, the use of certain drugs and damage to the pancreas are also causes of AP.^5^ Interestingly, pancreatic acinar cells after injury may secrete chemical factors, cytokines and various cell adhesion factors (tumour necrosis factor [TNF], interleukin [IL]‐6, IL‐18, IL‐1β, etc.) to induce the infiltration of immune cells, which contribute to the progression of AP.^6^ Despite achievements in care, imaging and interventional approaches, AP is still related to significant morbidity and mortality.^7^

Extracellular vesicles (EVs) are a heterogeneous family of membrane‐limited vesicles and can be internalized via endocytosis or membrane fusion, releasing their contents into ‘recipient’ cells.^8^ These EVs contain many proteins, sugars, lipids and multiple kinds of genetic materials, such as DNA, mRNA and non‐coding (nc) RNAs.^9^ Long non‐coding RNA (lncRNA) metastasis‐associated lung adenocarcinoma transcript‐1 (MALAT1) can act as competitive endogenous RNA (ceRNA) to regulate its downstream target genes via interaction of microRNA response elements (MREs) and microRNAs (miRNAs).^10^ Accumulating evidence proves that knockdown of MALAT1 promotes differentiation of macrophages into M1 subtypes in hepatocellular carcinoma.^11^ Additionally, MALAT1 shuttled by exosomes from oxidized low‐density lipoprotein‐treated endothelial cells can promote the M2 polarization of macrophages in cardiovascular disease.^12^ Depleted MALAT1 has been demonstrated to promote the M1 polarization of macrophages in inflammation of central nervous system.^13^ What's more, downregulation of MALAT1 could inhibit LPS‐induced activation of M1 macrophages and activates IL‐4 induced M2 differentiation.^14^ Recent study reveals that MALAT1 promotes AP through the miRNA‐194/YAP1 axis.^15^ Importantly, activated macrophages in AP can differentiate into pro‐inflammatory M1 subtypes and secrete some cytokines and regulatory factors to induce local inflammation of the pancreas, systemic inflammatory responses or damage to functions of multiple tissues.^16^, ^17^ However, the specific mechanisms of MALAT1‐mediated M1 polarization of macrophages remain unclear. This study attempts to explore how MALAT1 influences the occurrence of AP, and tries to reveal whether MALAT1 affects AP by regulating M1 polarization of macrophages.

## 2 MATERIALS AND METHODS

### 2.1 Ethic statement

All participants signed informed consent, and this study was performed with the approval of the Ethics Committee of the Second Affiliated Hospital of Xi'an Jiaotong University. This study was carried out in strict accordance with the *Declaration of Helsinki*. All animal experiments were approved by the laboratory animal care committee of Xi'an Jiaotong University. All experimental procedures that involved animals were approved according to the guidelines of the Care and Use of Laboratory Animals by the National Institute of Health, China.

### 2.2 Bioinformatic analysis

The AP‐related lncRNA expression microarray data set (GSE121038) was obtained from the GEO database (https://www.ncbi.nlm.nih.gov/geo/), including 4 normal samples and 4 AP samples. The ‘limma’ package of R language was utilized for differential analysis with normal samples as a control. The differentially expressed genes in AP were obtained with |logFC| >1, *p* value <0.05 as the threshold. The exoRBase database (http://www.exorbase.org/exoRBase/toIndex) was employed to retrieve the expression of MALAT1 in EVs. The starBase database (starbase.sysu.edu.cn/) was used to search the binding sites of MALAT1 and miR‐181a‐5p in humans and mice. Next, the starBase database and TargetScan database (http://www.targetscan.org/vert_71/) were utilized to predict the target genes of miR‐181a‐5p in humans and mice, and the mirDIP database (http://ophid.utoronto.ca/mirDIP/index.jsp#r) was used to further predict the target genes of miR‐181a‐5p in humans. The ‘clusterprofiler’ package of the R language was employed to perform KEGG pathway enrichment analysis on candidate target genes. The target binding sites of miR‐181a‐5p and high‐mobility group box 1 protein (HMGB1) in humans and mice were attained through the TargetScan database.

### 2.3 Study subjects

The serum samples (20–50 ml) were collected from 40 AP patients (average age of 57.23 ± 9.48 years old; 24 males and 16 females) and 40 healthy individuals (average age of 56.75 ± 8.95 years old, 22 males and 16 females). AP patients were diagnosed at the Second Affiliated Hospital of Xi'an Jiaotong University, and the patients had no other diseases. The detailed information of AP patients is shown in Table S1.

### 2.4 EV isolation

Serum samples (3 ml) were centrifuged at 2000 *g* (46962, Thermo Fisher) for 10 min and at 10,000 *g* for 30 min at 4℃. The obtained supernatant was resuspended in 8 ml phosphate‐buffered saline (PBS) and ultracentrifuged at 120,000 *g* for 70 min in a 30% sucrose buffer. The sucrose fraction was recovered, washed with PBS, filtered through a 0.22‐µm filter and ultracentrifuged again at 120,000 *g* for 70 min. Then, EV precipitate was resuspended by an appropriate amount of PBS for later use or frozen at −80℃.

### 2.5 Transmission electron microscope (TEM)

EV suspension (20 μl) was loaded onto formvar carbon‐coated copper electron microscopy grids for 2 min and then treated with phosphotungstic acid solution (12501‐23‐4, Sigma‐Aldrich Chemical Company, St Louis, MO, USA) for 5 min. Grids were washed three times with PBS to remove redundant phosphotungstic acid solution and then maintained in semi‐dry state. The images were observed under a TEM (Zeiss Inc., Thornwood, NY, USA).

### 2.6 Nanoparticle tracking analysis (NTA)

NTA was conducted using NanoSight analyser (NanoSight LM10‐HS; Malvern, Worcestershire, UK). Briefly, EVs (10–20 μl) were diluted with PBS to the final volume of 1 ml and 1 ml EVs were injected into the sample pool using a 1‐ml syringe. The focal length of the main engine knob was adjusted to see clear ‘white bright spot’, and the gain was adjusted for recording, 30 s per time. A small amount of sample was slowly injected into the sample pool using a syringe after 30 s of single recording, and the detection was repeated three times for each sample. The track of each EV in the screen was analysed and was automatically converted into the diameter and concentration of EVs according to the Brownian motion principle. The original concentration could be obtained via dilution ratio through conversion.

### 2.7 EV uptake assay

A PKH67 green fluorescence kit (MINI67‐1KT, Sigma‐Aldrich) was used to label purified EVs from human serum. EVs were resuspended in 1 ml Diluent C solution, and then, 4 μl PKH67 ethanol dye solution was added into Diluent C solution to prepare 4 × 10^−6^ M dye solution. Then, 1 ml EV suspension was mixed with PKH26 for 5 min and cultured with 2 ml of 1% bovine serum albumin (BSA) for 1 min to terminate staining. The labelled EVs were ultracentrifuged at 100,000 × *g* for 70 min at 4℃ and washed using PBS. EVs were ultracentrifugated again and resuspended in 50 μl PBS. PKH67‐labelled EVs were incubated with THP‐1 cells for 12 h. The cells were fixed with 4% paraformaldehyde and washed with PBS. The nuclei were stained with 4’,6‐diamidino‐2‐phenylindole (DAPI) (Sigma‐Aldrich). The uptake of labelled EVs was determined using a confocal microscope (Leica, Oskar Barnack, Germany).

### 2.8 Establishment of AP cell models

Mouse pancreatic acinar carcinoma cell line MPC‐83 cells (CL‐0518, Procell Life Science & Technology Co., Ltd., Wuhan, China) were incubated in Roswell Park Memorial Institute (RPMI)‐16401 medium (Gibco, Carlsbad, CA, USA) supplemented with 10% foetal bovine serum (FBS, HyClone Company, Logan, UT, USA) and 1% double antibody (Gibco) with saturated humidity and 5% CO_2_ at 37℃. After culturing for 24 h, MPC‐83 cells were incubated with 100 nM caerulein (#C9026, Sigma‐Aldrich). Then, the cells were harvested at 0, 4, 8, 12 or 24 h.

### 2.9 Extraction of MPC‐83 cell‐derived EVs

The MPC‐83 cells were passaged after recovery. When reaching 70% confluence, MPC‐83 cells were washed thrice with 0.01 mol/L PBS and cultured for 48 h in FBS medium without EVs. The supernatant was collected and centrifuged at 300 *g* and 2000 *g* for 10 and 30 min, respectively, to remove cells. Next, the supernatant was centrifuged at 10,000 *g* for 30 min to remove cell debris. The suspension was resuspended in sucrose buffer, centrifugated at 100,000 *g* (46962, Thermo Fisher) for 70 min, washed with PBS and centrifugated at 100,000 *g* for 70 min again. Then, EV precipitate was resuspended by an appropriate amount of PBS for later use or frozen at −80℃.

### 2.10 Establishment of M1 polarization models of macrophages

Mouse mononuclear macrophage RAW264.7 cells (Shanghai Cell Bank, Chinese Academy of Sciences, Shanghai, China) with good growth status were plated onto a 24‐well plate and stimulated into M1 cells with Dulbecco's modified Eagle's medium (DMEM) (Gibco) containing 20 ng/ml IFN‐γ, 100 ng/ml LPS and 10% FBS for 18 h. Subsequently, the cells were induced into M2 cell with DMEM (Gibco) containing 20 ng/ml IL‐4 and 10% FBS for 18 h. Macrophages cultured in DMEM containing the same amount of PBS and 10% FBS were used as unpolarized cell (M0). The levels of cell polarization‐related factors (inducible nitric oxide synthase [iNOS], IL‐6, TNF‐α, Arg1, and IL‐10) were determined by reverse transcription‐quantitative polymerase chain reaction (RT‐qPCR) to verify the polarization.

### 2.11 Cell transfection

MALAT1 small‐interfering RNA (si‐MALAT1), miR‐181a‐5p mimic, miR‐181a‐5p inhibitor, HMGB1 overexpression plasmid (oe‐HMGB1), si‐HMGB1 and the corresponding control plasmids were purchased from Guangzhou RiboBio Co., Ltd. (Guangzhou, China). The MALAT1 siRNA and control siRNA were transfected or co‐transfected into MPC‐83 or RAW264.7 cells using Lipofectamine 2000 reagent (Invitrogen, Carlsbad, California, USA), and miR‐181a‐5p mimic, miR‐181a‐5p inhibitor, oe‐HMGB1 and si‐HMGB1 were transfected into RAW264.7 cells.

### 2.12 RT‐qPCR

RNA was extracted using TRIzol reagent (Invitrogen). LncRNA and mRNA were reversely transcribed into complementary DNA (cDNA) using a PrimeScript™ RT Master Mix Kit (Takara Bio Inc., Otsu, Shiga, Japan). miRNA was reversely transcribed into cDNA with PolyA tailing using PolyA tailing detection kit (B532451, Shanghai Sangon Biotechnology Co. Ltd., Shanghai, China, containing universal PCR reverse primers). The primers of lncRNA, mRNA and miRNA were purchased from Guangzhou RiboBio. U6 and glyceraldehyde‐3‐phosphate dehydrogenase (GAPDH) were used as the internal reference for miRNA and lncRNA/mRNA, respectively. The 2^−ΔΔCt^ method was used to quantify the relative expression of target genes. The primers used are listed in Table S2.

### 2.13 Argonaute2 (AGO2) pull‐down assay

293T cells and MPC‐83 cells were transfected with miR‐NC or miR‐181a‐5p mimic, respectively. After transfection for 48 h, AGO2 pull‐down assay was performed with the transfected cells by using the Magna RIPTM RNA Binding Protein Immunoprecipitation Kit (Millipore, Bedford, MA, USA). The cells were incubated with anti‐AGO2 antibody (Millipore) or negative control IgG (Millipore), and then, the relative enrichment of MALAT1 and HMGB1 was measured by RT‐qPCR.

### 2.14 Western blot analysis

Cell was washed three times with PBS and lysed with radioimmunoprecipitation assay lysis +protease inhibitor (Roche) on ice for 30 min, followed by centrifugation at 15,000 *g* at 4℃ for 10 min. The protein concentration was measured by the BCA Assay Kit (#A53225, Thermo Fisher Scientific Inc., Waltham, MA, USA). Proteins were separated with sodium dodecyl sulphate‐polyacrylamide gel electrophoresis gel (10%) and then transferred onto a polyvinylidene difluoride membrane (Millipore). After blocked with 5% bovine serum albumin (BSA) for 2 h at 4℃, membranes were incubated with specific primary antibodies CD63 (1:1000, ab216130, Abcam Inc., Cambridge, UK), CD81 (1:1000, ab109201, Abcam), tumour susceptibility gene 101 (TSG‐101) (1:1000, ab125011, Abcam), calnexin (1:1000, ab22595, Abcam), HMGB1 (1:1000, Abcam), TLR4 (1:500, Abcam), nuclear factor‐kappaB (NF‐κB) (1:1000, ab32360, Abcam), I‐kappa‐B‐alpha (IKBa) (1:1000, ab32518, Abcam) and GAPDH (1:1000, #5174, Cell Signaling Technologies, Beverly, MA, USA) overnight at 4℃. The membranes were then washed with TBST three times (10 min per wash) and incubated with secondary antibody (1:5000) peroxidase‐conjugated AffiniPure goat anti‐rabbit IgG (H + L) (#111035003, Jackson ImmunoResearch, USA) or peroxidase‐conjugated AffiniPure goat anti‐mouse IgG (H + L) (#115035003, Jackson ImmunoResearch, USA) for 1 h. The immunocomplexes on the membrane were visualized using enhanced chemiluminescence (Thermo Fisher Scientific) on chemiluminescence instrument (ChemiDoc XRS+, Bio‐Rad, Richmond, Cal., USA).

### 2.15 Northern blot analysis

A total of 30 μg of the indicated RNA was subjected to formaldehyde gel electrophoresis and then transferred to a Biodyne nylon membrane (Pall, NY). Biotin (Roche, Mannheim, Germany)‐labelled MALAT1 cRNA probe was prepared using in vitro transcription from pSPT19‐MALAT1 with the probe sequence of ACGAATTCAGGGTGAGGAAGTAAAAACAGGTCATCTATTCACAAAACTGA. Radio‐labelled DNA oligonucleotides were regarded as detection probes for Northern blot detection of miRNA. For molecular markers, the Decade Marker System (Ambion) was used, and the probe sequence was ACTCACCGACAGCGTTGAATGTT. After pre‐hybridization in ULTRAhyb buffer (Ambion, Grand Island, NY) for 60 min, the membrane was hybridized, washed and tested as described above.

### 2.16 Dual‐luciferase reporter gene assay

Fragments of MALAT1‐wild type (WT) and MALAT1‐mutation (MUT), HMGB1‐WT and HMGB1‐MUT were synthesized and then inserted into the pmirGLO vector (Invitrogen). The above‐constructed plasmids were co‐transfected with miR‐NC or miR‐181a‐5p mimic into the 293T cells (Shanghai Cell Bank, Chinese Academy of Sciences). After transfection for 48 h, the luciferase activity was measured with Dual‐Luciferase Reporter Assay System Kit (Promega Corporation, Madison, WI, USA).

### 2.17 Animal experiments

A total of 32 healthy male C57BL/6J mice (6–8 weeks) were purchased from Beijing Vital River Laboratory Animal Technology Co., Ltd. (Beijing, China). C57BL/6 mice were kept in a specific pathogen‐free animal facility with humidity of 60–65%, temperature of 22–25℃ and given free access to food and water under a 12‐h light and dark cycle. Mice were acclimated 1 week before the experiment, and the health of the mice was observed before the experiment.

To establish AP mouse models, mice were intraperitoneally injected with 12 times of caerulein (50 μg/kg), once every one hour. Mice were divided into 4 groups, control (blank control), AP (AP mice injected with saline), AP +sh‐NC (AP mice injected with sh‐NC) and AP +sh‐MALAT1 (AP mice injected with sh‐MALAT1). In vivo shRNA was purchased from Guangzhou RiboBio Co., Ltd. In order to silence the expression of MALAT1 in mouse pancreas, in vivo shRNA‐MALAT1 (15 nmol/20 g) was injected through the common bile duct before intraperitoneal administration.

### 2.18 Enzyme‐linked immunosorbent assay (ELISA)

The cell supernatant or serum of AP mice was collected to detect the TNF‐α and IL‐6 according to the instructions of TNF alpha Mouse ProQuantum Immunoassay Kit (A43658, Thermo Fisher Scientific) and IL‐6 Mouse ProQuantum Immunoassay Kit (A43656, Thermo Fisher Scientific) though microplate reader (Invitrogen; Thermo Fisher Scientific, Inc.).

### 2.19 Immunohistochemical staining

One hour after the last injections of caerulein, the mice were sacrificed with the blood from the inferior vena cava extracted and pancreatic tissues separated. The samples were then fixed, dehydrated and made into paraffin‐embedded sections. According to the instructions of immunohistochemistry kit (# SA1054, BOSTER Biological Technology Co. Ltd., Wuhan, Hubei, China), the sections were subjected to retrieval repair with the citric acid antigen, washed thrice with PBS, blocked at room temperature for 20 min and incubated with the primary antibody at 4℃ overnight. The next day, the sections were cultured with the secondary antibody at room temperature for 45 min. Finally, the sections were stained with DAB and the nucleus was stained with haematoxylin. The images were observed and photographed under a microscope (Olympus Optical Co., Ltd, Tokyo, Japan). Myeloperoxidase‐Anti (MPO) antibody was purchased from Abcam (1:500, ab208670).

### 2.20 Haematoxylin and eosin (H&E) staining

The paraffin‐embedded tissues were deparaffinized, gradient hydrated and stained with haematoxylin (#AR‐0711, Beijing Dingguo Changsheng Biotechnology Co., Ltd, Beijing, China) for 1 – 2 min. Next, the sections were stained with eosin (#AR‐0731, Beijing Dingguo Changsheng Biotechnology), followed by gradient dehydration and sealing. The images were observed and photographed under a microscope (Olympus Optical Co., Ltd, Tokyo, Japan). The extent of cell injury and necrosis was quantified through computer‐aided morphological examination by experienced morphologists.

### 2.21 Detection of serum amylase and lipase

Serum amylase and lipase were the most common serum markers in AP, which may reflect the severity of AP. After extraction of blood, the lipase detection kit (#A054‐1–1, NanJing JianCheng Bioengineering Institute, Nanjing, China) and the amylase detection kit (#BC0615, Beijing Solarbio Science & Technology Co. Ltd., Beijing, China) were used to detect the activity of lipase and amylase in the serum of mice though microplate reader (Invitrogen; Thermo Fisher Scientific, Inc.).

### 2.22 Flow cytometry

Peritoneal lavage was used to isolate peritoneal macrophages, and flow cytometry was used to analyse the phenotype of peritoneal macrophages. Briefly, peritoneal macrophages were washed with staining buffer (1% BSA in PBS containing 0.01% NaN_3_, Thermo Fisher, USA) and incubated with 10% mouse serum for 20 min on ice. Subsequently, the cells were incubated with reagents from the LIVE/DEAD™ Fixable Dead Cell Stain Kit (Thermo Fisher), FITC‐conjugated anti‐CD163 (Bio‐Rad) and PE‐conjugated anti‐CD86 (BD Biosciences, Franklin Lakes, NJ, USA) at the manufacturer's recommended dilution for 40 min on ice. For intracellular staining, the cells were fixed and permeabilized with fixation buffer from a Fixation/Permeabilization Solution Kit (BD Biosciences) for 1 h at 4℃ in the dark, washed with permeabilization buffer, incubated with Alexa‐Flour647‐conjugated anti‐CD68 (Bio‐Rad) antibody and treated with permeabilization buffer for 1 h at 4℃ in the dark. The samples were then washed and resuspended in permeabilization buffer and analysed with a BD Accuri TM C6 Plus (BD Biosciences). The results were analysed with the BD FACS DIVA software (BD Biosciences).

### 2.23 Immunofluorescence staining

The distribution of the M1 phenotype in the pancreas was assayed by immunofluorescence staining. Pancreatic tissues were cut into sections which were dewaxed. CD68 (ab955, Abcam) expression was detected by anti‐mouse Alexa‐Flour647 (P0191, Biotechnology Co., Shanghai, China), and CD86 (ab119857, Abcam) expression was detected by anti‐rabbit FITC (Beyotime). Then, the samples were stained with DAPI to visualize the nuclei. The distribution of M1 macrophages in the pancreas was examined by a fluorescence microscope (Olympus Optical Co., Ltd, Tokyo, Japan).

### 2.24 Statistical analysis

Data analysis was performed using the SPSS 21.0 software (IBM, Armonk, NY, USA). All measurement data are presented as mean ±standard deviation. Unpaired *t* test was used for comparisons of independent samples between two groups. For multiple group comparisons, one‐way analysis of variance (ANOVA) and Tukey's post hoc tests were used. A value of *p* < 0.05 was considered significant.

## 3 RESULTS

### 3.1 MALAT1/miR‐181a‐5p/HMGB axis is involved in the occurrence and development of AP

**FIGURE 1:**
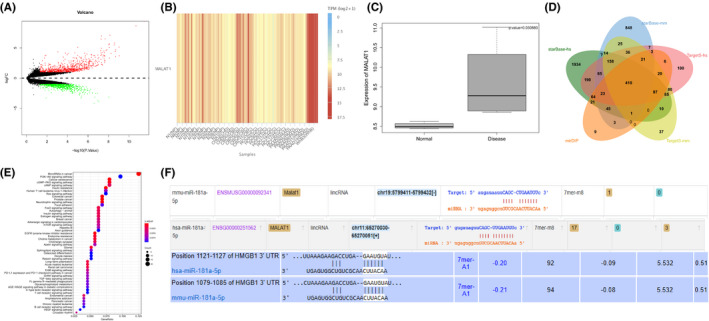
The expression and binding relationship of MALAT1/miR‐181a‐5p/HMGB axis in AP samples. A, Volcano map of differentially expressed genes in AP‐related microarray data set GSE121038. The abscissa represents log 10pvalue, and the ordinate represents log FC. The red points in the Figure represent the significantly upregulated genes in AP, and the green points represent the significantly downregulated genes in AP. B, Expression of MALAT1 in EVs. The abscissa indicates the source of the EVs, and the histogram on the right is the colour scale (NP, normal person; CHD, coronary heart disease; CRC, colorectal cancer; HCC, hepatocellular carcinoma; PAAD, pancreatic adenocarcinoma; WhB, whole blood). C, Expression of MALAT1 in the microarray data set GSE121038. The abscissa represents the sample type, and the ordinate represents the expression value. The left box plot represents normal samples, and the right box plot represents disease samples. The upper right corner is the differential p value. D, Prediction of target genes of miR‐181a‐5p. The five ellipses in the Figure represent the prediction results of target genes in humans and mouse using starBase and TargetScan databases, and the prediction results of target genes in humans using the mirDIP database. The middle part represents the intersection of five sets of data. E, KEGG pathway enrichment analysis of candidate target genes, wherein the abscissa represents GeneRatio, and the ordinate represents the KEGG entry. The size of the circle in the Figure represents the number of enriched genes in the entry, the colour indicates *p* value of the enrichment, and the histogram on the right is the colour scale. F, Binding site information of MALAT1, miR‐181a‐5p and HMGB1 in humans and mice

Differential analysis of AP‐related lncRNA microarray data set GSE121038 yielded 1549 differentially expressed genes (Figure 1A). Through further gene type annotation, we found that there were 3 lncRNAs (Table S3); among them, we found highly expressed MALAT1 in serum‐derived EVs through exoRBase (Figure 1B). At the same time, MALAT1 was found highly expressed in AP samples in the GSE121038. Further prediction revealed that MALAT1 was capable of binding to miR‐181a‐5p in humans and mouse (Figure 1C).

The starBase database was used to predict target genes of miR‐181a‐5p in humans and mouse, and 410 candidate target genes were obtained after intersection of prediction results (Figure 1D). Through KEGG pathway enrichment analysis (Figure 1E), we found that 410 candidate target genes were mainly enriched in PI3K‐AKT signalling pathway and animal autophagy‐related pathways.

Existing studies indicate that regulation of autophagy can affect macrophage polarization.^18^, ^19^ In the autophagy pathway, there was a candidate target gene HMGB1. Accumulating evidence demonstrates that HMGB1 regulates TLR4 expression to facilitate AP occurrence and development, and TLR4 promotes M1 polarization of macrophages.^20^, ^21^, ^22^ In humans and mouse, there were binding sites among MALAT1, miR‐181a‐5p and HMGB1 (Figure 1F). Thus, it can be concluded that the involvement of the MALAT1/miR‐181a‐5p/HMGB axis affects the occurrence and development of AP.

### 3.2 MALAT1 is upregulated in pancreatic cell‐derived EVs and serum EVs in AP patients

**FIGURE 2:**
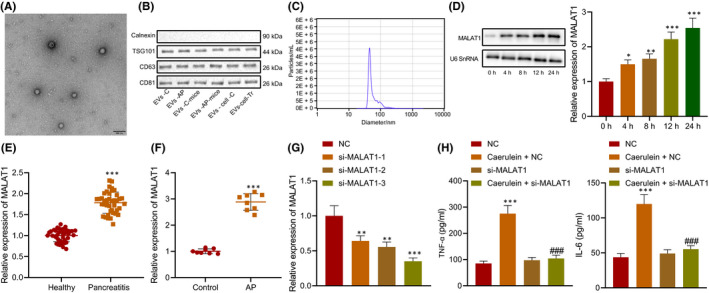
Highly expressed MALAT1 in MPC‐83 cell‐derived EVs and EVs from serum of AP patients. A, EVs in serum and cell supernatant detected by TEM. B, Expression of EV markers, calnexin (EVs‐negative), TSG‐101, CD63 and CD81 (EVs‐positive) measured by Western blot analysis. C, Particle size of EVs analysed by NTA. D, MALAT1 expression in MPC‐83 cells determined using RT‐qPCR and Northern blot analysis. E, MALAT1 expression in the serum of AP patients and healthy normal people (*n* = 40) determined using RT‐qPCR. F, MALAT1 expression in the serum of AP mice and normal mice (*n* = 8) determined using RT‐qPCR. G, MALAT1 interference efficiency detected by RT‐qPCR. H, Levels of IL‐6 and TNF‐α in the MPC‐83 cells treated with si‐MALAT1. * vs. 0 h, healthy normal people, normal mice or MPC‐83 cells treated with NC. # vs. MPC‐83 cells treated with si‐MALAT1. * or # *p* < 0.05. ** or ## *p* < 0.01. *** or ### *p* < 0.001. Data are expressed as the mean ± standard errors

MALAT1 is reported to induce AP progression.^15^ In addition, EVs can aggravate the inflammatory responses of AP mice and cells.^23^ To determine the expression of MALAT1 in serum of AP patients or pancreatic cell‐derived EVs, the EVs were isolated from the plasma of AP patients (EVs‐AP), the plasma of healthy volunteers (EVs‐C), plasma of AP mouse models (EVs‐AP‐mice), plasma of normal mice (EVs‐C‐mice), supernatant of untreated MPC‐83 (EVs‐cell‐C) and supernatant of caerulein‐treated MPC‐83 cells (EVs‐cell‐Tr). TEM exhibited that the separated EVs had a double‐layer membrane structure with a diameter between 40 and 200 (Figure 2A). Meanwhile, Western blot analysis revealed the exist of positive markers of EVs, CD81, CD63 and TSG‐101 in EVs, but no negative marker Calnexin (Figure 2B). NTA analysis showed that the diameter of EVs was about 50–150 nm (Figure 2C). These results suggested successful isolation of EVs.

MPC‐83 cells were treated with caerulein to induce AP cell models. The expression of MALAT1 in the cell supernatant was detected at 0, 4, 8, 12 and 24 h using RT‐qPCR and Northern blot analysis. With the increasing time of caerulein treatment, the expression of MALAT1 also increased, showing a time‐dependent manner (Figure 2D). Determination of MALAT1 expression in the serum of AP patients and AP mice showed elevated MALAT1 expression in the serum of AP patients and AP mice (Figure 2E,F).

Next, si‐MALAT1‐1, si‐MALAT1‐2 and si‐MALAT1‐3 were designed, and all of them significantly reduced MALAT1 expression (Figure 2G). The si‐MALAT1‐3 with the highest interference was selected for subsequent experiments. MPC‐83 cells were transfected with si‐MALAT1 and treated with caerulein. ELISA exhibited that silencing of MALAT1 reduced the levels of inflammatory factors in the MPC‐83 cells (Figure 2H). The above results indicated that MALAT1 was highly expressed in pancreatic cell‐derived EVs and serum‐derived EVs of AP patients, and silencing MALAT1 inhibited the levels of inflammatory factors in MPC‐83 cells.

### 3.3 EVs containing MALAT1 promote M1 polarization of macrophages

**FIGURE 3:**
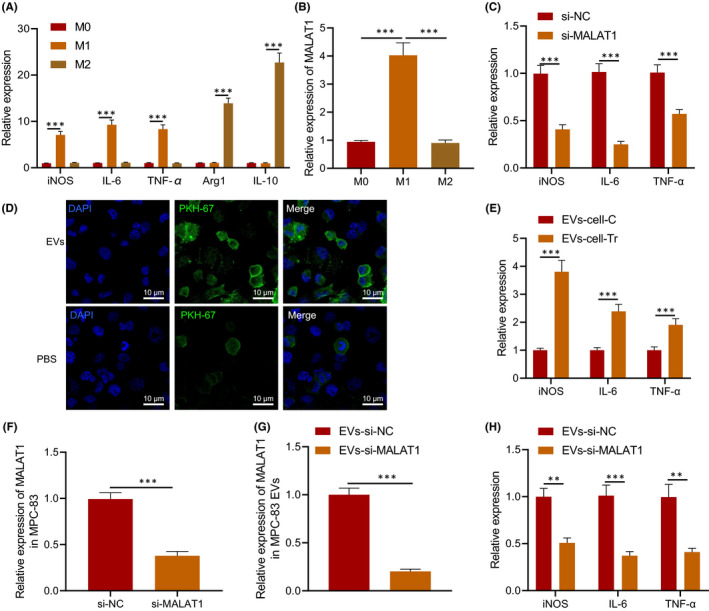
EV‐encapsulated MALAT1 enhanced the M1 polarization of macrophages. A, The levels of related factors in M0, M1 and M2 macrophages determined using RT‐qPCR. B, MALAT1 in macrophages determined using RT‐qPCR. RAW264.7 monocytes were transduced with si‐MALAT1. C, Levels of iNOS, IL‐6 and TNF‐α in RAW264.7 cells determined using RT‐qPCR. D, Uptake of EVs by RAW264.7 cells. PKH67‐labelled EVs are green fluorescence, and DAPI‐stained nucleus is blue fluorescence, scale bar = 10 μm. RAW264.7 cells were co‐cultured with EVs extracted from caerulein‐treated MPC‐83 cells. E, Levels of M1 markers in RAW264.7 cells. MPC‐83 cells were transduced with si‐MALAT1. F, MALAT1 expression in MPC‐83 cells determined using RT‐qPCR. G, Expression of EVs‐secreted MALAT1 in MPC‐83 cells determined using RT‐qPCR. H, Levels of M1 markers detected in RAW264.7 cells co‐treated with MPC‐83 cells. * *p* < 0.05. ** *p* < 0.01. *** *p* < 0.001. Data are shown as the mean ± standard errors

It has been found that M1 polarization of macrophages aggravates AP.^24^ Our previous experimental results also revealed that MALAT1 exerted an important effect on AP. Therefore, we tried to analyse whether EVs‐MALAT1 could affect the occurrence and development of AP by affecting the M1 polarization of macrophages. RAW264.7 monocytes were subjected to M1 and M2 polarization induction, and the cells and supernatant were collected. RT‐qPCR (Figure 3A) showed that compared with M0 macrophages, mRNA levels of M1 polarization markers, such as iNOS, IL‐6 and TNF‐α, elevated in IFN‐γ‐ and LPS‐induced RAW264.7 monocytes; besides, mRNA levels of M2 polarization markers, Arg1 and IL‐10, also increased in IL‐4‐induced RAW264.7 monocytes, indicating that the M1 polarization models of M1 and M2 macrophages were successfully constructed. RT‐qPCR also exhibited that MALAT1 in M1 macrophages was higher than that in M0 and M2 macrophages, suggesting that MALAT1 was related to M1 polarization of macrophages (Figure 3B).

To explore the effect of MALAT1 on the M1 polarization of macrophages, RAW264.7 monocytes were transfected with si‐MALAT1 and induced to M1 polarization by IFN‐γ and LPS. It was found that silencing of MALAT1 reduced the levels of iNOS, IL‐6 and TNF‐α (Figure 3C).

To verify the effect of EVs on the M1 polarization of macrophages in AP, MPC‐83 cells were treated with caerulein followed by extraction of EVs. PKH67‐labelled EVs were co‐cultured with RAW264.7 for 12 h, followed by fixation and photograph. The results displayed that EVs could be internalized by RAW264.7 cells (Figure 3D). RAW264.7 cells were then co‐cultured with EVs extracted from caerulein‐treated MPC‐83 cells. After induction to M1 polarization, levels of iNOS, IL‐6 and TNF‐α in RAW264.7 cells were higher in EVs from caerulein‐treated MPC‐83 cells than those without caerulein treatment (Figure 3E). When MPC‐83 cells were transfected with si‐MALAT1 (Figure 3F), EVs with downregulated MALAT1 were extracted (Figure 3G) with the findings uncovered that compared with EVs‐si‐NC, levels of iNOS, IL‐6 and TNF‐α decreased in RAW264.7 cells treated with EVs‐si‐MALAT1 (Figure 3H). The obtained data confirmed that EVs containing MALAT1 induced M1 polarization of macrophages.

### 3.4 MALAT1 upregulates HMGB1 expression by competitively binding to miR‐181a‐5p

**FIGURE 4:**
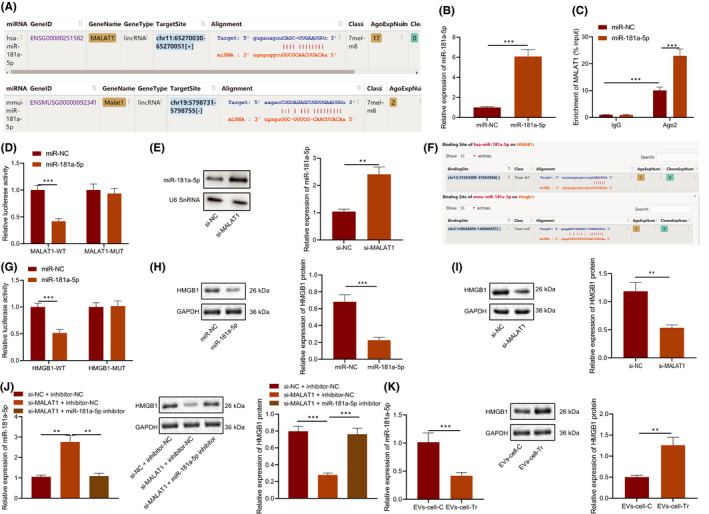
MALAT1 regulated HMGB1 by competitively binding to miR‐181a‐5p. A, Binding sites between MALAT1 and miR‐181a‐5p predicted using bioinformatics analysis. RAW264.7 cells were transduced with miR‐181a‐5p mimic. B, miR‐181a‐5p expression in RAW264.7 cells determined using RT‐qPCR. C, The binding site between MALAT1 and miR‐181a‐5p verified by AGO2 pull‐down assay. D, The targeted binding of MALAT1 to miR‐181a‐5p verified using the dual‐luciferase reporter gene assay. E, The expression of miR‐181A‐5p after MALAT1 silencing detected by RT‐qPCR and Northern blot analysis. F, Binding sites between miR‐181a‐5p and HMGB1 predicted using bioinformatic analysis. G, The targeted binding of miR‐181a‐5p to HMGB1 verified using the dual‐luciferase reporter gene assay. RAW264.7 cells were transduced with miR‐181a‐5 mimic. H, HMGB1 protein level in RAW264.7 cells measured using Western blot analysis. RAW264.7 cells were transduced with si‐MALAT1. I, HMGB1 protein level in RAW264.7 cells measured using Western blot analysis. RAW264.7 cells were transduced with miR‐181a‐5 inhibitor and si‐MALAT1. J, HMGB1 protein level in RAW264.7 cells measured using Western blot analysis. RAW264.7 cells were treated with EVs from caerulein‐treated MPC‐83 cells. K, miR‐181a‐5p expression and HMGB1 protein level in RAW264.7 cells measured using RT‐qPCR and Western blot analysis, respectively. *p < 0.05. **p < 0.01. ***p < 0.001. Data are shown as the mean ± standard errors

The results of bioinformatic analysis showed that MALAT1 could bind to miR‐181a‐5p (Figure 4A). As a negative regulator of AP, miR‐181a‐5p could repair AP damage.^25^ AGO2 pull‐down assay presented (Figure 4B,C) that the enrichment degree of MALAT1 in complex pulled down by AGO2 was higher in RAW264.7 cells treated with miR‐181a‐5p mimic than in cells without treatment. Dual‐luciferase reporter gene assay exhibited that luciferase activity of MALAT1‐WT was inhibited by miR‐181a‐5p mimic (*p* < 0.05), while no evident difference was found in MALAT1‐MUT (*p* > 0.05) (Figure 4D). The results of RT‐qPCR and Northern blot analysis showed that depleted MALAT1 upregulated miR‐181a‐5p expression, indicating that MALAT1 negatively regulated miR‐181a‐5p expression (Figure 4E).

There was a binding site between miR‐181a‐5p and 3'UTR of HMGB1 through online software analysis (Figure 4F). Prior study confirmed that HMGB1 was capable of facilitating AP.^20^ Further luciferase assay exhibited that luciferase activity of HMGB1‐WT was inhibited by miR‐181a‐5p mimic (*p* < 0.05), while no evident difference was found in HMGB1‐MUT (*p* > 0.05) (Figure 4G).

HMGB1 protein level decreased in RAW264.7 cells treated with miR‐181a‐5p mimic (Figure 4H) or with si‐MALAT1 (Figure 4I). Meanwhile, compared with RAW264.7 cells transfected with si‐NC +inhibitor‐NC, miR‐181a‐5p expression increased and HMGB1 protein level reduced in RAW264.7 cells transfected with si‐MALAT1 + inhibitor‐NC; compared with RAW264.7 cells transfected with si‐MALAT1 + inhibitor‐NC, miR‐181a‐5p expression decreased and HMGB1 protein level increased in RAW264.7 cells transfected with si‐MALAT1 + miR‐181a‐5p inhibitor (Figure 4J). Moreover, miR‐181a‐5p expression also decreased and HMGB1 protein level increased in RAW264.7 cells treated with EVs from caerulein‐treated MPC‐83 cells (Figure 4K). It can be concluded that MALAT1 regulated HMGB1 expression by competitively binding to miR‐181a‐5p.

### 3.5 EV‐encapsulated MALAT1 promotes the M1 polarization of macrophages by upregulating HMGB1 and activating the TLR4/NF‐κB signalling pathway

**FIGURE 5:**
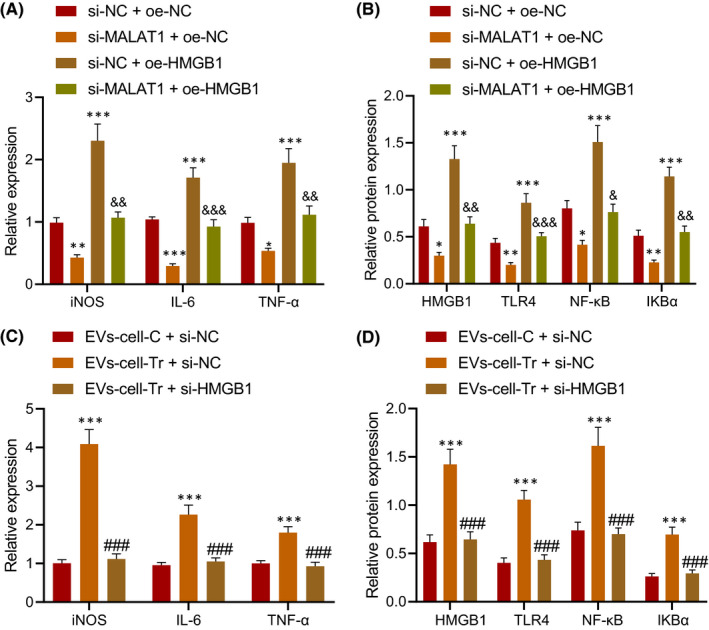
EV‐encapsulated MALAT1 induced the M1 polarization of macrophages by upregulating HMGB1 and activating the TLR4/NF‐κB signalling pathway. RAW264.7 cells were transduced with si‐MALAT1 and/or oe‐HMGB1 plasmids, and induced to M1 polarization after transfection for 24 h. A, Levels of iNOS, IL‐6 and TNF‐α in RAW264.7 cells. B, Levels of HMGB1, TLR4, NF‐κB and IKBa in RAW264.7 cells measured by Western blot analysis. RAW264.7 cells were treated with EVs from caerulein‐treated MPC‐83 cells and transduced with si‐HMGB1. C, Levels of iNOS, IL‐6 and TNF‐α in RAW264.7 cells. D, Levels of HMGB1, TLR4, NF‐κB and IKBa in RAW264.7 cells measured by Western blot analysis. * *vs*. RAW264.7 cells treated with EVs‐cell‐C + si‐NC or si‐NC +oe‐NC; # *vs*. RAW264.7 cells treated with EVs‐cell‐Tr +si‐NC. & *vs*. RAW264.7 cells treated with si‐MALAT1 + oe‐NC. *, # or & *p* < 0.05. **, ## or && *p* < 0.01. ***, ### or &&& *p* < 0.001. Data are shown as the mean ± standard errors

To investigate the effect of upregulated HMGB1 expression on M1 polarization of macrophages, RAW264.7 cells were transfected with NC, si‐MALAT1 and oe‐HMGB1 plasmids, respectively, and induced to M1 polarization after transfection for 24 h (Figure S1). The results displayed that silencing of MALAT1 inhibited the levels of iNOS, IL‐6 and TNF‐α in RAW264.7 cells, while the results were opposite in RAW264.7 cells transfected with oe‐HMGB1, and RAW264.7 cells transfected with si‐MALAT1 and oe‐HMGB1 showed similar levels of iNOS, IL‐6 and TNF‐α as the RAW264.7 cells treated with NC (Figure 5A).

It has been reported that HMGB1 can regulate the occurrence and development of AP through the TLR4/NF‐κB signalling pathway.^20^ Through Western blot analysis, we found reduced protein levels of HMGB1, TLR4, NF‐κB and IKBa in RAW264.7 cells treated with si‐MALAT1, and they were elevated in RAW264.7 cells transfected with oe‐HMGB1, while levels of HMGB1, TLR4, NF‐κB and IKBa were recovered in RAW264.7 cells transfected with si‐MALAT1 and oe‐HMGB1 (Figure 5B).

In addition, RAW264.7 cells were treated with EVs from caerulein‐treated MPC‐83 cells and transfected with si‐HMGB1. ELISA (Figure 5C) and Western blot analysis (Figure 5D) exhibited that levels of iNOS, IL‐6, TNF‐α and protein levels of HMGB1, TLR4, NF‐κB and IKBa increased in RAW264.7 cells treated with EVs from caerulein‐treated MPC‐83 cells, while opposite results were found in RAW264.7 cells treated with EVs from caerulein‐treated MPC‐83 cells and transduced with si‐HMGB1. These findings proved that MALAT1 shuttled by EVs promoted HMGB1 expression and activated the TLR4/NF‐κB signalling pathway to promote M1 polarization of macrophages.

### 3.6 Silencing of MALAT1 attenuates pancreatic tissue injury in AP mice

**FIGURE 6:**
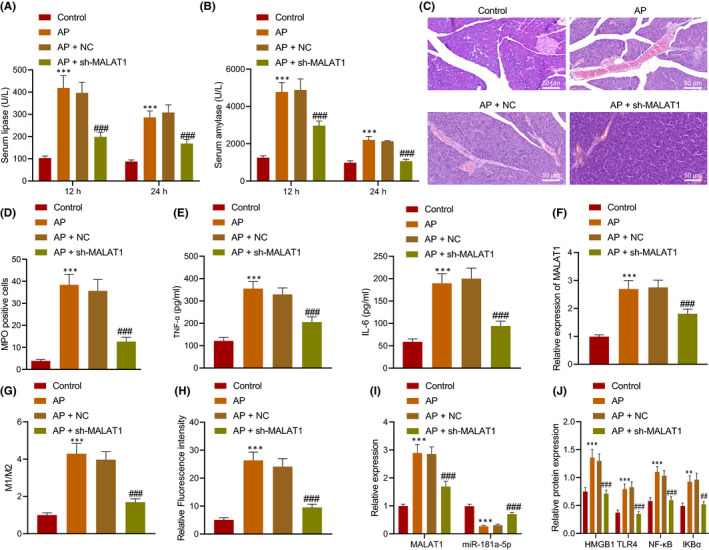
Silencing of MALAT1 reduced pancreatic tissue injury in AP mice. AP mice were injected with sh‐MALAT1 (*n* = 8). A, Determination of serum amylase and lipase in AP mice. B, Determination of lipase in AP mice. C, Pancreatic tissue morphology in AP mice detected by H&E staining, scale bar = 25 μm. D, MPO expression in pancreatic tissues of AP mice. E, Levels of IL‐6 and TNF‐α in pancreatic tissues of AP mice measured by ELISA. F, MALAT1 expression in the serum‐derived EVs determined using RT‐qPCR. G, The phenotype of peritoneal macrophages detected by flow cytometry. H, The M1/M2 ratio detected by flow cytometry. H, M1 macrophages in pancreatic tissues detected by immunofluorescence staining. I, Expression of MALAT1 and miR‐181a‐5p in the pancreas of AP mice determined by RT‐qPCR. J, Protein levels of HMGB1, TLR4, NF‐κB and IKBa in the pancreas of AP mice determined by Western blot analysis. * *vs*. normal mice; # *vs*. AP mice or AP mice injected with NC. * or # *p* < 0.05. ** or ## *p* < 0.01. *** or ### *p* < 0.001. Data are shown as the mean ± standard errors

AP mouse models were established to explore the effects of MALAT1 on the occurrence and development of AP in vivo. It was found that levels of serum amylase and lipase expression significantly increased in serum of AP mice, and they decreased in AP mice injected with sh‐MALAT1 (Figure 6A,B). Observation from H&E staining exhibited that pancreatic tissues in AP mice showed mesenchymal congestion, oedema, inflammatory cell infiltration, focal or confluent necrosis and haemorrhage (Figure 6C). Immunohistochemistry (Figure 6D), ELISA (Figure 6E) and RT‐qPCR (Figure 6F) revealed that levels of MPO, IL‐6 and TNF‐α, and MALAT1 increased in pancreatic tissues of AP mice, while levels of MPO, IL‐6 and TNF‐α, and MALAT1 reduced in pancreatic tissues of AP mice injected with sh‐MALAT1. The obtained data suggested that downregulation of MALAT1 inhibited the release of inflammatory factors to relieve pancreatic tissue injury in AP mice.

To further demonstrate the effect of MALAT1 on macrophages in vivo, the polarization phenotype of peritoneal macrophages was assayed by flow cytometry. CD68 acted as the marker of all macrophages, CD68^+^CD86^+^ macrophages were identified as M1 macrophages, and CD68^+^CD163^+^ macrophages were identified as M2 macrophages. Flow cytometry showed that M1 macrophages and the M1/M2 ratio increased in the AP mice, and M1 macrophages and the M1/M2 ratio decreased after injection of sh‐MALAT1 (Figure 6G). Meanwhile, immunofluorescence staining displayed that the number of M1 macrophages elevated in pancreas of AP mice, but reduced in pancreas of AP mice injected with sh‐MALAT1 (Figure 6H). RT‐qPCR and Western blot analysis revealed that the levels of MALAT1, HMGB1, TLR4, NF‐κB and IKBa increased and miR‐181a‐5p reduced in pancreas of AP mice, while the trends were reversed in pancreas of AP mice injected with sh‐MALAT1 (Figure 6I). Taken together, MALAT1 promoted pancreatic tissue injury in AP by affecting M1 polarization of macrophages *in vivo*, and silencing of MALAT1 reduced pancreatic tissue injury in AP.

## 4 DISCUSSION

In the present study, we found that MALAT1 was highly expressed in the serum, pancreatic tissues and pancreatic cells of patients with AP as well as pancreatic cell‐derived EVs, which indicated that MALAT1 may negatively regulate the occurrence and development of AP. Moreover, EV‐encapsulated MALAT1 promotes the M1 polarization of macrophages by upregulating HMGB1 and activating the TLR4/NF‐κB signalling pathway.

Existing research reveals that aberrant expression of MALAT1 plays a critical role in the occurrence of pancreatitis.^26^ Our findings demonstrated highly expressed MALAT1 in EVs of AP cell models, indicating that MALAT 1 could be encapsulated by EVs. The increased expression of MALAT1 in serum‐derived EVs of patients with AP revealed that MALAT1 in AP patients could be wrapped into the EVs and secreted out, which was consistent with the results in vitro, and MALAT1 was highly expressed in the serum of patients with AP. The above‐mentioned results proved the important role of MALAT1 in the occurrence and development of AP. The use of exosomes as natural carriers for therapeutic delivery of miRNA, siRNA, mRNA and lncRNA has generated great interests.^27^ A recent study noted highly expressed MALAT1 in pancreatic cancer cells and tissues as well as exosomes derived from serum of pancreatic ductal adenocarcinoma patients.^28^ Besides, the regulatory role of MALAT1 in the occurrence and development of AP through the miR‐194/YAP1 axis has been confirmed by previous study.^15^ Gu et al. have reported that upregulation of MALAT1 in serum may be due to the secretion of pancreatic tissue into serum.^15^

In addition, the obtained data from our study suggested that the EVs carrying MALAT1 could promote the M1 polarization of macrophages, which indicated that the EVs could be captured by the macrophages, and the MALAT1 shuttled by EV could be released into the macrophages to regulate the specific gene expression of macrophages and promote their M1 polarization. A prior study has demonstrated that MALAT1 knockdown represses the LPS‐induced M1 polarization of macrophages and promotes IL‐4‐induced M2 polarization of macrophages.^14^ siRNA‐mediated knockdown of MALAT1 promotes M1 polarization of macrophages.^13^ Oxidized low‐density lipoprotein (oxLDL)‐treated endothelial cells containing MALAT1 enhance M2 polarization of macrophages.^12^ Moreover, downregulated MALAT1 inhibits LPS‐induced TNF‐α and IL‐6 secreted by macrophages to suppress the LPS‐induced M1 polarization of macrophages.^29^ These findings support that MALAT1 can regulate the polarization of macrophages, but the specific molecular mechanism needs more in‐depth study.

Furthermore, the present results showed that MALAT1 could regulate the expression of HMGB1 by competitively binding to miR‐181a‐5p. Interestingly, exosomes carry multiple kinds of lncRNAs, which can regulate gene expression by translational suppression or by acting as competitive endogenous RNA.^30^ Several studies have documented that MALAT1 regulates HMGB1 expression by inhibiting miR‐370‐3p, miR‐129‐5p or miR‐181c‐5p.^31^, ^32^, ^33^ Our findings demonstrated that MALAT1 upregulated HMGB1 in macrophages, while HMGB1 overexpression activated the TLR4/NF‐κB signalling pathway to promote the M1 polarization of macrophages, thereby regulating the occurrence of AP. Jia et al. confirmed that downregulation of MALAT1 alleviated myocardial inflammatory damage through the miR‐26a/HMGB1/TLR4/NF‐κB axis.^34^ Moreover, in vivo experimental results proved that silencing of MALAT1 could reduce pancreatic tissue damage in mice with AP, which was consistent with the in vitro experimental results, providing a theoretical basis and possible therapeutic targets for the treatment of AP.

**FIGURE 7:**
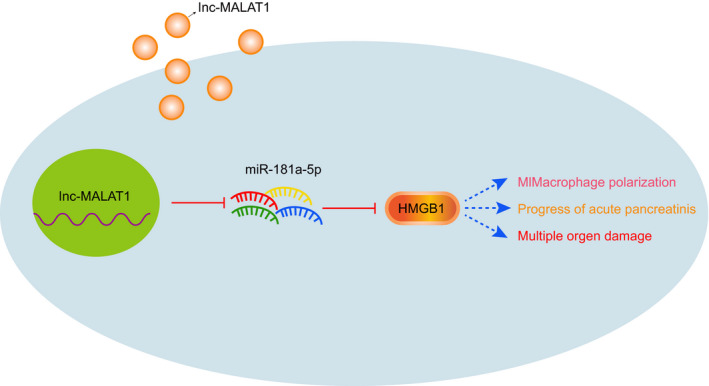
The molecular mechanism of MALAT1 shuttled by EVs in affecting M1 polarization of macrophages involved in the occurrence and development of AP via miR‐181a‐5p/HMGB1 axis

To sum up, EV‐encapsulated MALAT1 upregulated the HMGB1 expression and activated the TLR4 signalling pathway by competitively binding to miR‐181a‐5p which facilitated the M1 polarization of macrophages and ultimately promoted the occurrence and progression of AP (Figure 7). Although there have been many studies on the role of MALAT1 on AP as well as the fact that MALAT1 regulates HMGB1 through various miR,^15^, ^32^, ^33^, ^35^ it remains elusive how MALAT1 regulates AP in AP through specific miRNAs. The current study highlighted that EVs encapsulated MALAT1 and systematically revealed that MALAT1 regulated HMGB1 through miR‐181‐5p. In addition, MALAT1 could regulate the macrophages polarized to M1 phenotype in AP, but the specific molecular mechanism warranted further investigation and discussion.

## CONFLICT OF INTEREST

The authors declare that they have no conflict of interests.

## AUTHOR CONTRIBUTIONS

**Jie Liu:** Conceptualization (equal); Formal analysis (equal); Investigation (equal); Writing‐original draft (equal); Writing‐review & editing (equal). **Zequn Niu:** Conceptualization (equal); Investigation (equal); Writing‐original draft (equal); Writing‐review & editing (equal). **Rui Zhang:** Conceptualization (equal); Methodology (equal); Writing‐original draft (equal); Writing‐review & editing (equal). **Zhuo Peng:** Formal analysis (equal); Investigation (equal); Writing‐review & editing (equal). **Liming Wang:** Data curation (equal); Formal analysis (equal); Methodology (equal); Writing‐review & editing (equal). **Zhong Liu:** Data curation (equal); Formal analysis (equal); Investigation (equal); Writing‐review & editing (equal). **Yanxia Gao:** Conceptualization (equal); Formal analysis (equal); Investigation (equal); Writing‐review & editing (equal). **Honghong Pei:** Formal analysis (equal); Investigation (equal); Methodology (equal); Writing‐review & editing (equal). **Longfei Pan:** Conceptualization (equal); Funding acquisition (equal); Investigation (equal); Writing‐review & editing (equal).

## Supporting information

Fig S1

Table S1‐S3

## Data Availability

The data sets generated and/or analysed during the current study are available from the corresponding author on reasonable request.
