# Supplementary material for: MALAT1 shuttled by extracellular vesicles promotes M1 polarization of macrophages to induce acute pancreatitis via miR‐181a‐5p/HMGB1 axis
Source: J Cell Mol Med. 2021 Aug 27;25(19):9241–54. doi: 10.1111/jcmm.16844 (PMC8500974; doi:10.1111/jcmm.16844)
Supplement: Supplementary file 1 — Fig S1 [file JCMM-25-9241-s001.docx]

**
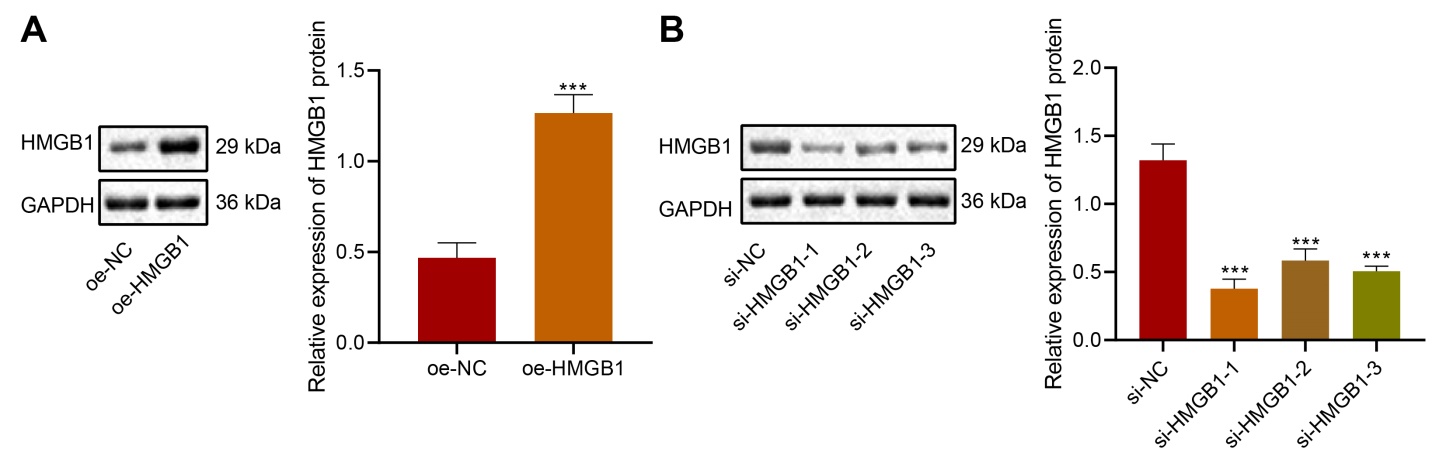
**

**FIGURE S1** Verification of transfection efficiency. A, Overexpression efficiency of HMGB1 in RAW264.7 cells verified by Western blot analysis. B, Knockdown efficiency of HMGB1 in RAW264.7 cells verified by Western blot analysis. * *vs.* RAW264.7 cells transfected with oe-NC or si-NC. * *p* < 0.05, ** *p* < 0.01, *** *p* < 0.001. Data are shown as the mean ± standard errors.
