# Supplementary material for: MALAT1 shuttled by extracellular vesicles promotes M1 polarization of macrophages to induce acute pancreatitis via miR‐181a‐5p/HMGB1 axis
Source: J Cell Mol Med. 2021 Aug 27;25(19):9241–54. doi: 10.1111/jcmm.16844 (PMC8500974; doi:10.1111/jcmm.16844)
Supplement: Supplementary file 2 — Table S1‐S3 [file JCMM-25-9241-s002.docx]

**Table S1** Clinical characteristics of the included AP patients

| Characteristics | AP (n = 40) |
| --- | --- |
| Sex |  |
| Female | 16 |
| Male | 24 |
| Age (years) | 57.23 ± 9.48 |
| Etiology |  |
| Biliary disease | 12 |
| Alcoholism | 10 |
| Hypertriglyceridemia | 7 |
| Other | 11 |
| Ranson score |  |
| < 3 | 15 |
| ≥ 3 | 25 |
| Smoking history | 20.3 ± 4.8 |
| Lipase (U/l) | 428.34 ± 13.92 |
| Amylase (U/l) | 463.28 ± 15.46 |

**Table S2** RT-qPCR primer sequences

| Genes | Gene sequences |
| --- | --- |
| TNF-α | F 5’-CTGAACTTCGGGGTGATCGG-3’ |
|  | R 5’-GGCTTGTCACTCGAATTTTGAGA-3’ |
| miR-181a-5p | F 5’-AACATTCAACGCTGgTCGGTGAGT-3’ |
| iNOS | F 5’-GGAGTGACGGCAAACATGACT-3’ |
|  | R 5’-TCGATGCACAACTGGGTGAAC-3’ |
| IL-6 | F 5’-TGGTCTTCTGGAGTACCATAGC-3’ |
|  | R 5’-TGTGACTCCAGCTTATCTCTTGG-3’ |
| Arg1 | F 5’-TTGGGTGGATGCTCACACTG-3’ |
|  | R 5’-GTACACGATGTCTTTGGCAGA-3’ |
| IL-10 | F 5’-GCTCTTACTGACTGGCATGAG-3’ |
|  | R 5’-CGCAGCTCTAGGAGCATGTG-3’ |
| **HMGB1** | F 5’-CCAAGAAGTGCTCAGAGAGGTG-3’ |
|  | R 5’-GTCCTTGAACTTCTTTTTGGTCTC-3’ |
| U6 | F 5’-AAAGCAAATCATCGGACGACC-3’ |
|  | R 5’-GTACAACACATTGTTTCCTCGGA-3’ |
| Human-GAPDH | F 5’-GTCTCCTCTGACTTCAACAGCG-3’ |
|  | R 5’-ACCACCCTGTTGCTGTAGCCAA-3’ |
| Mouse-GAPDH | F 5’-GGGTCCCAGCTTAGGTTCAT-3’ |
|  | R 5’-CTCGTGGTTCACACCCATCA-3’ |
| Human-MALAT1 | F 5’-AGCTCTGTGGTGTGGGATTG-3’ |
|  | R 5’-GTGGCAAAATGGCGGACTTT-3’ |
| Mouse-MALAT1 | F 5’-GGACTTGAGCTGAGGTGCTT-3’ |
|  | R 5’-TCCTAGCTTCCTCCACCGAA-3’ |

Note: RT-qPCR, Reverse transcription quantitative polymerase chain reaction; TNF-α, Tumor necrosis factor-α; miR-181a-5p, microRNA-181a-5p; iNOS, inducible nitric oxide synthase; IL-6, interleukin-6; IL-10, interleukin-10; **HMGB1, high mobility group protein 1;** GAPDH, glyceraldehyde-3-phosphate dehydrogenase; MALAT1, metastasis-associated lung adenocarcinoma transcript 1.

**Table S3** Gene type annotation

| symbol | logFC | AveExpr | P.Value |
| --- | --- | --- | --- |
| NEAT1 | 2.899409031 | 14.57862298 | 6.57E-07 |
| SNHG3 | 1.395502875 | 10.90837153 | 0.000406669 |
| MALAT1 | 1.094645344 | 9.061104984 | 0.030880907 |

NEAT1, nuclear paraspeckle assembly transcript 1; SNHG3, Small nucleolar RNA host gene 3; MALAT1, metastasis-associated lung adenocarcinoma transcript 1.
